# Late onset Wilson Disease with normal neuro-psychiatric status: A case report

**DOI:** 10.1016/j.amsu.2022.103678

**Published:** 2022-05-06

**Authors:** Bishal Dhakal, K.C. Prabhat, Abinash Karki, Ayush Mohan Bhattarai, Sachin Sapkota, Binaya Subedi, Abhinav Dahal

**Affiliations:** a Nepalese Army Institute of Health Sciences, Sanobharyang, Kathmandu, Nepal; b Maulakalika Hospital Pvt. Ltd., Bharatpur, 10-Chitwan, Nepal

**Keywords:** Wilson disease, Chronic liver disease, Kayser-Fleischer ring, Leipzig scoring

## Abstract

**Introduction:**

Late onset Wilson disease (WD) is a rare form of WD. WD has variability of clinical presentations from acute liver failure to chronic liver disease (CLD). The hepatic and neurological variants of WD have wider variations.

**Case presentation:**

A 55-year-old female, known case of CLD, presenting with generalized body swelling and abdominal pain, was diagnosed with late onset WD with normal neuro-psychiatric status. She was treated with zinc and considered for liver transplantation.

**Clinical discussion:**

Late onset WD is itself a rare form of WD. Within it, neurological manifestations are common in late onset WD, which was quite opposite as compared to our case. Similarly, diagnostic delay has been a concern in late onset WD with CLD as with our case.

**Conclusions:**

In spite of being uncommon in later age, WD and its different variations like with normal neuro-psychiatric status should be considered as an etiology in cases of unexplained liver diseases.

## Highlights

• Wilson disease (WD) has been one of the causes for chronic liver disease (CLD) but late onset WD is one of the rare forms in WD.
• Neurological manifestations are common in late onset WD which is absent in our case.
• There is common diagnostic delay of WD in later ages with chronic liver disease because of variability in presentations of WD.
• The treatment for WD has been updated but based on the availability d-penicillamine or zinc has been a common choice.

## 1 Introduction

Wilson disease (WD), an autosomal recessive variant, is a multisystem disorder affecting liver primarily. It affects brain and osseo-muscular system progressively [1]. It is classically described as “progressive lenticular degeneration” by Dr. Samuel Alexander Kinnier Wilson [2,3]. Although WD is commonly seen in children and younger adult population, there are many reported cases of WD diagnosed at older age [1,4]. Usually, the hepatic presentation of WD is common in children and younger population. And as the age increases, the neurological presentation of WD is common (4). The cases of late onset neurological variant-WD without Kayser–Fleischer (KF) ring have been reported in literature [5,6]. But cases of late onset WD with normal neuro-psychiatric status have been scarce in medical literature. Hence, this is a case of late onset WD in a 55-year-old female with normal neurological status.

## 2 Case presentation

A 55-year-old Hindu female presented to the emergency department of our center with generalized body swelling and abdominal pain. The pain was burning type mostly located towards epigastric region. It was not associated with nausea, vomiting, waterbrash and diarrhea. She was a diagnosed case of chronic liver disease (CLD) for four years whose etiology was unknown till then. She gave negative history for alcohol consumption and use of liver toxic drugs. There was no family history of liver disease. There was jaundice present in whole body, pallor and bilateral pitting pedal edema on general examination. Her vitals were stable at admission. On per abdominal examination, the abdomen was distended with flanks full. There was generalized tenderness and shifting dullness was present. There were no signs of hepatic encephalopathy (HE). The higher mental function and rest of central nervous system examinations were normal.

The baseline investigations are shown in Table 1.

**Table 1:** Baseline investigations (TIBC: Total Iron Binding Capacity; CRP-C: C-Reactive Protein-C).

Laboratory tests	Result	Unit	Reference range
Total Leukocytes Count	3.7	10^˄^3/μL	4–11
Neutrophil	55	%	40–80
Lymphocyte	37	%	20–40
Hemoglobin	10.3 (L)	g/dl	13–17
Platelet Count	90 (L)	10^˄^3/μL	150–450
Red Blood Cell (RBC) Count	3.36	10˄6/μL	4.5–5.5
Urea	29	mg/dl	17–43
Creatinine	0.9	mg/dl	0.7–1.3
Sodium	141	mEq/L	135–145
Potassium	3.7	mEq/L	3.5–5.5
Bilirubin Total	2.3 (H)	mg/dl	0.1–1.2
Bilirubin Direct	1.2 (H)	mg/dl	0.0–0.2
Amylase	52	U/L	40–140
Alkaline Phosphatase (ALP)	85	U/L	53–128
Alanine Transferase (ALT)	32	U/L	0–35
Aspartate Transferase (AST)	65 (H)	U/L	0–35
Random Blood Glucose	108.8	mg/dl	70–140
Prothrombin time (PT)	19.3 (H)	seconds	11–13.5
International Normalized Ratio (INR)	1.4 (H)		0.8–1.1
Troponin I	Negative		
Urine Routine Examination	Normal		
Lactate Dehydrogenase (LDH)	204	U/L	140–280
Total Cholesterol	157	mg/dl	<200
Triglyceride	98	mg/dl	70–150
Serum Iron	117.47	μg/dl	50–100
Ferritin	687.2	μg/L	20–250
CRP-C	Negative		
TIBC	139	μg/dl	228–428

As from clinical examination and investigations, she presented as CLD with decompensating features like ascites, jaundice and coagulopathy. The ascitic fluid analysis showed total count of 100 cells/cubic mm and discrete leukocyte count of 80% lymphocytes and 20% neutrophils. The patient was not in spontaneous bacterial peritonitis (SBP). Similarly, ultrasound of abdomen and pelvis revealed, coarse echotexture of liver parenchyma with irregular outline suggestive of CLD and moderate ascites. The previously done esophago-gastro-duodenoscopy showed small esophageal varices. These investigations were done to confirm the liver pathology and to evaluate the etiologies for chronic liver disease as presented by the patient. He was diagnosed as CLD-Child Pugh C with decompensating features and anemia of chronic disease (ACD) from iron profile studies.

As etiology for CLD was unidentified, etiological workup was done for the differentials like chronic viral hepatitis, WD, alcoholic liver disease and non-alcoholic fatty liver disease. The etiological workup is given as follows Table 2.

**Table 2:** Etiological workup.

Serology	Non-reactive for HIV, HBsAg and HCV
Alcoholic history	Negative
Lipid profile and blood sugar	Within Normal Limits
Serum Ceruloplasmin (CPN)	10.80 mg/dl (Normal range: 20–60)
24 h urinary copper	436.24 μg/day (Normal range: 3–50) 229.60 μg/L (Normal range: 2–80)
Ophthalmology consultation	Kayser–Fleischer (KF) seen in both eye

**Fig. 1:**
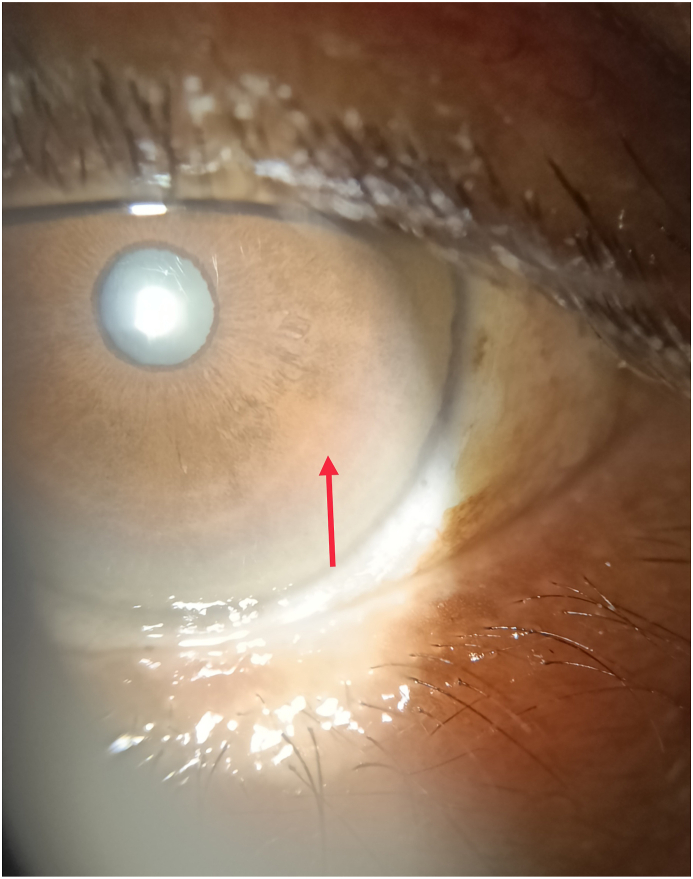
KF ring in eye Legend: Arrow shows KF ring in eye.

**Fig. 2:**
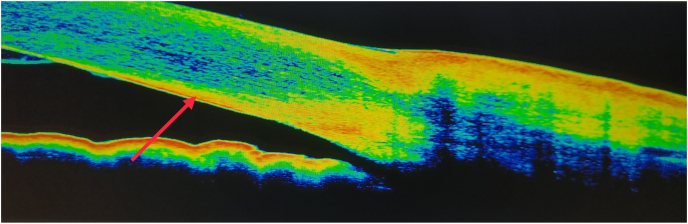
KF ring in Optical Coherence Tomography Legend: Arrow shows Hyperintense shadow seen in the stroma of cornea most likely deposits of copper (KF ring at the level of Descemet membrane).

The classical finding Kayser–Fleischer (KF) ring is shown in Fig. 1 and Fig. 2. After the etiological workup was done, she was diagnosed as Wilson disease (WD) based on American Association for the Study of Liver Diseases (AASLD). Simultaneously, based on Leipzig scoring, diagnosis of WD was established as the score was 5. There were no neurological features present on the case.

She was treated with tablet Zinc 50 mg BD (twice a day). As she was stable on general examination and vital parameters, she was discharged on zinc 50 mg BD with the advice for follow up in medical OPD after 1 month.

## 3 Discussion

Wilson disease has been a disorder of wide variations and presentations. Over the years from its first description (1912), the spectrum of WD has been ever changing. The newer concepts and understanding about the disease are still evolving. As WD has been described as a rare disease, the prevalence of disease was found to be between 1.2/100,000 and 2.0/100,000 in European countries (7). And the average prevalence worldwide was estimated to be around 30 per million population [8]. It has been exceeded by recent genetic studies as 142 per million population [9]. The majority of patients fall between age 5 and 35 but the variations can be seen from as early as 9 months of age to ninth decade [9,10].

**Fig. 3:**
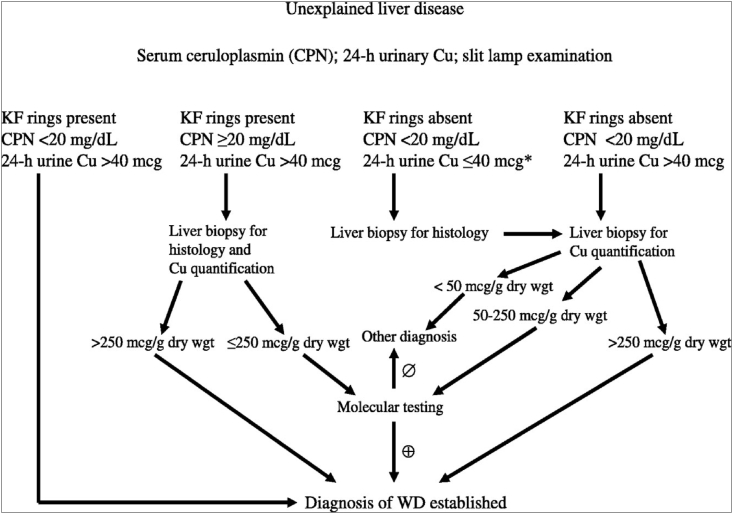
Approach to diagnosis of Wilson disease in a patient with unexplained liver disease from the “AASLD Practice Guidelines—Diagnosis and Treatment of Wilson Disease: An Update.” Legend: Reproduced from Roberts EA and Schilsky ML. Diagnosis and treatment of Wilson disease: An update. Hepatology 2008:47; 2089–2111. https://doi.org/10.1002/hep.22261 with permission from John Wiley and Sons.

The mean age with hepatic and neurological presentations in WD has been described to be 11 years and about 15–21 years respectively [7]. The neurologic variant frequency increases with age. But variations are found among these findings too which pose challenge in diagnosis of WD at earlier stage. Wilson disease is an inborn error in copper metabolism due to the mutation in ATP7B gene, located on chromosome 13. The diagnostic criteria for WD has been suggested by few guidelines like American Association for the Study of Liver Diseases (AASLD) using clinical and biochemical algorithm and European Association for the Study of the Liver (EASL) using Leipzig scoring system [[11], [12], [13]]. The algorithms for diagnosis of WD by AASLD and Leipzig scoring system are shown in Fig. 3 and Table 3 respectively.

**Table 3:** Scoring system developed at the 8th International Meeting on Wilson's disease, Leipzig 2001.

Typical clinical symptoms and signs	Other tests
**KF rings** •Present 2 •Absent 0	**Liver copper (in the absence of cholestasis)** •>5x ULN (>4 umol/g) 2 •0.8-4 μmol/g 1 •Normal (0.8 umol/g) −1 •Rhodamine-Positive granules 1
**Neurological Symptoms** • Severe 2 • Mild 1 • Absent 0	**Urinary copper (in the absence of acute hepatitis)** •Normal 0 •1-2x ULN 1 •>2x ULN 2 •Normal, but >5x ULN after d- penicillamine 2
**Serum Ceruloplasmin** • Normal (>0.2 g/L) 0 • 0.1–0.2 g/L 1 • < 0.1 g/L 2	**Mutation analysis** •On both chromosomes detected 4 •On 1 chromosome detected 1 •No mutations detected 0
**Coombs-negative hemolytic anemia** • Present 1 • Absent 0	
**Total score**	**Evaluation**
1) 4 or more Diagnosed established 2) 3 Diagnosed possible, more tests needed 3) 2 or less Diagnosis very unlikely	

Legend: Reproduced from Ferenci P, Czlonkowska A, Stremmel W, Houwen R, Rosenberg W, Schilsky M et al. EASL Clinical Practice Guidelines: Wilson's disease. Journal of Hepatology. 2012; 56(3):671–85. https://doi.org/10.1016/j.jhep.2011.11.007 with permission from Elsevier.

On the basis of AASLD, the diagnosis was established as WD in our case as it met all the three criteria for its diagnosis in unexplained liver disease [8]. The three criteria are presence of KF ring, serum CPN <20 mg/dl and 24-h urine copper >40 mcg. Similarly, based on EASL guideline using Leipzig scoring, she was diagnosed as WD as the diagnosis would be established when the score come to be 4 or more [12]. The score was 5 which included presence of KF ring, serum CPN level 0.1–0.2 g/L and 24-h urinary copper >2 × upper limit of normal (ULN).

The hepatic manifestations in WD include acute liver failure, chronic liver disease (compensated or decompensated), asymptomatic elevation of alanine transferase (ALT) or aspartate transferase (AST), non-alcoholic fatty liver disease, acute hepatitis and hepatocellular carcinoma. Similarly, neurological presentations are dystonia, tremor, dysarthria, ataxia, parkinsonism, chorea and peripheral neuropathy. Several other involvements include psychiatric, ocular, cardiac, endocrinologic, hematologic, renal, musculoskeletal and cutaneous features [9]. As neurological features increase with age and late onset WD is rare [8,14], our case was diagnosed as late-onset WD with normal neuropsychiatric status. Similarly, there was delay of 4 years in diagnosis of WD with hepatic presentations from the time of onset of early hepatic symptoms. There were no any neuropsychiatric features as described above. This was a typical finding in accordance with the literature showing longer delay from onset of symptoms until definite diagnosis of WD with neuropsychiatric symptoms than that of WD with hepatic symptoms [15,16].

Although our patient had CLD with decompensating features while the diagnosis of WD was made, it was not severe enough to consider for immediate liver transplantation as described in literature [9]. She was treated on Zinc at first as she was stable on general examination. The consideration for liver transplantation was put on hold until the response of Zinc therapy. As described in literature, the prognosis is variable for decompensated CLD in WD [9]. It was our limitation that we could not assess the prognosis because of inadequate follow up.

There were certain limitations in our article like inability to perform liver biopsy and gene sequencing due to unavailability of facility in our center. We could not do follow up with the patient to assess the long-term course of symptoms, progress of the therapy and prognosis of the disease. In addition, we also could not perform screening of family members for WD due to unavailability of the facility.

## 4 Conclusions

The discussion and amendments in the spectrum of WD are never ending topics. In the amidst of 21st century the variations in WD are being reported and published in a significant amount. We have reported such a variation of WD in our case report as late-onset WD diagnosed as decompensated CLD with normal neuropsychiatric status. It was an incidental finding during the etiology workup for unexplained liver disease. Though neuropsychiatric features are common with increasing age in WD, it will be wise to consider it even if the central nervous system (CNS) examination is normal, as it was in our case. This case report adds onto the current understanding of WD and its variability which may be useful to all the clinicians around the world in recognizing WD in a more diverse way.

## Ethical approval

This is a case report, therefore, it did not require ethical approval from ethics committee.

## Funding

The study did not receive any grant from funding agencies in the public, commercial or not-for-profit sectors.

## Author contributions

Author 1: Contributed in data collection, literature review, writing the manuscript.

Author 2: The resident physician, who helped in the diagnosis and supervised in case presentation.

Author 3: The resident physician, who helped in revising the manuscript.

Author 4: Contributed in manuscript review and data collection.

Author 5: Contributed in supervision and literature review.

Author 6: Contributed to literature review and data collection.

Author 7: Contributed in preparing manuscript and editing it.

All the authors read and approved the final manuscript.

## Declaration of interest statement

The authors report no conflicts of interest.

## Registration of research studies

Not applicable.

## Guarantor

Bishal Dhakal, Shree Birendra Hospital, 44600 Kathmandu, Nepal. Email: swarnimdhakal@gmail.com, Phone: +977 9846491651.

## Consent

Written informed consent was obtained from the patient for publication of this case report and accompanying images. A copy of the written consent is available for review by the editor-in-chief of this journal on request.

## Author agreement statement

We the undersigned declare that this manuscript is original, has not been published before and is not currently being considered for publication elsewhere.

We confirm that the manuscript has been read and approved by all named authors and that there are no other persons who satisfied the criteria for authorship but are not listed. We further confirm that the order of authors listed in the manuscript has been approved by all of us.

We understand that the Corresponding Author is the sole contact for the Editorial process. He/she is responsible for communicating with the other authors about progress, submissions of revisions and final approval of proofs.
